# Altered expression of caveolin 2 and 3 in smooth muscle of rat urinary bladder by 17β-estradiol

**DOI:** 10.1186/1471-2490-13-44

**Published:** 2013-09-16

**Authors:** Sun-Ouck Kim, Seung Hee Song, Seung-Chul Lee, Kyung Aa Cho, Jong Sung Park, Dongdeuk Kwon, Kwangsung Park

**Affiliations:** 1 Department of Urology, Research Institute of Medical Sciences, Chonnam National University, 8, Hak-dong, Dong-ku, Gwangju 501-757, Korea; 2 Department of Dermatology, Research Institute of Medical Sciences, Chonnam National University, Gwangju, Korea; 3 Department of Biochemistry, Research Institute of Medical Sciences, Chonnam National University, Gwangju, Korea; 4 Department of Physiology, Research Institute of Medical Sciences, Chonnam National University, Gwangju, Korea

**Keywords:** Caveolin, Bladder, Estrogen, Rat

## Abstract

**Background:**

The purpose of this study was to investigate the effect of estrogen alteration on the expression of caveolin 2 and 3 in rat smooth muscle of urinary bladder.

**Methods:**

Female Sprague–Dawley rats were divided into three groups: control, bilateral ovariectomy (Ovx), and bilateral ovariectomy followed by subcutaneous injections of 17β-estradiol (Ovx?+?Est). After 4 weeks, urodynamic measurements were taken to ascertain the contraction interval and contraction pressure. The expression and cellular localization of caveolin 2 and 3 were determined by Western blot and immunohistochemistry in rat urinary bladder smooth muscle.

**Results:**

In cystometrograms, the contraction interval (min) was significantly lower in the Ovx group (3.1?±?1.5) than in the control group (5.6?±?1.2), but was increased after estrogen treatment (9.3?±?1.0). Conversely, the average contraction pressure (mmHg) was higher in the Ovx group (26.2?±?2.3) than in the control group (21.9?±?3.1), and was decreased after estrogen treatment (23.8?±?3.5). Caveolin 2 and 3 expression was localized in the cell membrane of the smooth muscle. The protein expression of both caveolin 2 and 3 was significantly lower after ovariectomy and was restored to the control levels after 17β-estradiol treatment.

**Conclusions:**

Hormonal alteration causes a significant change in the expression of caveolin 2 and 3 in smooth muscle of rat urinary bladder. These findings suggest that these molecules might have functional roles in the detrusor overactivity that occurs in association with hormonal alteration.

## Background

Caveolae are small invaginations of the cell membrane that are membrane attached vesicular organelles present in most cell types, and which are particularly abundant in smooth muscle cells [1]. Caveolae are thought to play an important role in cell surface signaling and intracellular lipid transport [2]. Caveolins are major constituents of caveolae. Among them, caveolin 2 is expressed in many cell types, whereas caveolin 3 is the specific isoform that is present in skeletal and smooth muscle cells [3]. A complex and diverse functional role of caveolin has been proposed to provide a protective platform that regulates various signal molecules and may facilitate and integrate the cellular response to the specific extracellular stimulus [4,5]. Previously we investigated the effect of hormonal alterations on the expression of caveolin 1 in the urinary bladders of ovariectomized rats and reported that hormonal alterations cause a significant change in the expression of caveolin 1, which suggests that caveolin 1 might have a functional role in the overactivity of the detrusor muscle related to hormonal alterations in the urinary bladders [6]. Estrogen withdrawal induces a pronounce change in aged rat urinary bladder smooth muscle and estrogen replacement increases caveolar number and caveolinl protein [7].

Estrogen is essential for the physiologic function of the female lower urinary tract, which is believed to play an important role in bladder function [8,9]. Menopause or surgical removal of the ovary can result in lower urinary tract symptoms, and estrogen treatment for these subjects can induce pronounced alterations in these symptoms from the estrogen depletion [10,11]. No studies to date have investigated the changes in expression of caveolin 2 and caveolin 3 in the bladder smooth muscle of ovariectomized rats or the functional activity of these proteins in response to hormonal alteration.

The present study investigated the impact of estrogen on the expression of caveolin 2 and caveolin 3 of smooth muscle in rat urinary bladder. The specific aims of the present study were to examine how the protein expression of caveolin 2 and caveolin 3 changed in estrogen-depleted or replaced smooth muscle, and whether the altered density of caveolins correlated with altered contractile response in the rat urinary bladder smooth muscle. Understanding the effect of caveolin 2 and 3 on bladder smooth muscle under the hormonal change of estrogen could provide valuable insight into the mechanism regulating bladder overactivity and could identify caveolin protein as a promising target for the treatment of bladder dysfunction or bladder overactivity that occurs in association with the menopausal state in women.

## Methods

### Experimental model

Female Sprague–Dawley rats (12 weeks old, 230–240 g, N?=?45) were divided into three groups: control (Con, N?=?15), bilateral ovariectomy (Ovx, N?=?15), and bilateral ovariectomy plus subcutaneous injection with 17β-estradiol (Sigma-Aldrich, St. Louis, MO, USA) (Ovx?+?Est, N?=?15). The Con group underwent a sham operation. The Ovx group underwent a bilateral ovariectomy with intramuscular injection of zolazepam/tiletamine cocktail (4.4 mg/kg) and was treated with a subcutaneous injection of an oil vehicle. The Ovx?+?Est group underwent a bilateral ovariectomy, followed by treatment with subcutaneous estradiol daily (50 ug/kg/day) from 7 days after ovariectomy. All experimental animals were fed a standard diet up until the day before the experiment. Four weeks after ovariectomy and 3 weeks after hormonal replacement, animals with an estrous cycle confirmed via a vaginal smear were anesthetized with an intramuscular injection of a zolazepam/tiletamine cocktail (4.4 mg/kg) for tissue dissection after the cystometry. The study was approved by the Ethics Committee of Chonnam National University Medical School.

### Cystometrogram

Four weeks after the ovariectomy, 5 rats in each group were anesthetized with 1.2 g/kg urethane injected subcutaneously. A suprapubic midline incision was performed to expose the bladder, a transvesical catheter with a fire-flared tip (polyethylene catheter-50) was inserted into the dome of the bladder and secured with a ligature, and the abdomen was closed. The catheter was connected to a pressure transducer and syringe pump via a 3-way stopcock to record intravesical pressure and to infuse saline into the bladder. After the bladder was emptied, cystometry was performed with saline infused at a rate of 0.04 ml/min. The contraction pressure and contraction interval were recorded.

### Immunofluorescence staining

The tissue sections (N?=?5 in each group, eight sections in each tissue) were rinsed in phosphate-buffered saline (PBS), and then treated with 3% hydrogen peroxide in 60% methanol for 30 min to quench endogenous peroxidase activity. After washing in PBS, tissue sections were treated with normal chicken serum for 30 min to block nonspecific binding. After being washed in PBS, the sections were incubated with a 1:50 dilution in PBS of antibody for caveolin 2 or caveolin 3 (Chemicon, Temecula, CA, USA) for 12–14 h at 4°C. Immunoreactivity for caveolin 2 and caveolin 3 were detected with the use of Alexa-Fluor 594 chicken anti-mouse IgG (1;100, H?+?L; Invitrogen, Carlsbad, CA, USA). Tissues were mounted with the use of a mounting solution containing 4-6-diamidino-2-phenylindole. For a negative control, tissues were prepared in a similar manner, except that caveolin 2 and caveolin 3 were omitted from the incubation solution. Tissues were examined with a model LSM 510 confocal microscope (Carl Zeiss, Jena, Germany) with an excitation wavelength appropriate for Alexa-Fluor (405 or 594 nm). Final images were constructed with the use of LSM Image Examiner software.

### Western blot

All minced tissues were homogenized in ice-cold isolation solution with a Tissumizer homogenizer (Tekmar, Cincinnati, OH, USA). Tissues were homogenized with five bursts of five strokes of a micro-sawtooth generator. Tissue homogenates (N?=?5 in each group, 30 μg of protein) were separated by 12% sodium dodecyl sulfate-polyacrylamide gel electrophoresis and transferred to polyvinylidene fluoride membranes (Amersham Pharmacia Biotech, England, UK). The blots were then washed with Tris-Buffered Saline Tween (10 mM Tris–HCl, pH 7.6, 150 mM NaCl, 0.05% Tween-20). Each membrane was blocked with 5% skimmed milk for 1 h and incubated with the appropriate primary antibody. Monoclonal rabbit antibodies for caveolin 2 and caveolin 3 (1;1500, Chemicon) and polyclonal rabbit antibody against glyceraldehyde 3-phosphate dehydrogenase (GAPDH) (1;4000, Cell Signaling Technology, Danvers, MA, USA) were used. The membrane was then washed, and caveolin-2, caveolin-3, and GAPDH were detected with goat anti-mouse-IgG and goat anti-rabbit-IgG conjugated to horseradish peroxidase, respectively. Antibody incubations were performed in a 4°C incubator. The bands were visualized by enhanced chemiluminescence (Amersham Pharmacia Biotech, Buckinghamshire, UK). GAPDH was used as an internal control. Densitometry analysis was performed with a Studio Star Scanner using NIH image V1-57 software.

### Statistical analysis

The results are expressed as mean?±?standard deviation, except for the data for the cystometric parameters, which are expressed as mean?±?standard error of the mean. Analysis of variance was used to test the null hypothesis that there would be no differences in the mean expression levels between the three groups. Differences were considered significant at P?<?0.05.

## Results

All animals survived for 4 weeks after surgery. Body weight (g) was significantly higher in the Ovx group (362.3?±?10.1) than in the control group (267.1?±?11.5)(P?=?0.01). Treatment of ovariectomized animals with 17β-estradiol reduced the body weight to the control level (285.6?±?13.2)(P?=?0.03). There was no significant difference in bladder weight between the groups.

### Effect of estrogen on the cystometric parameters

**Figure 1:**
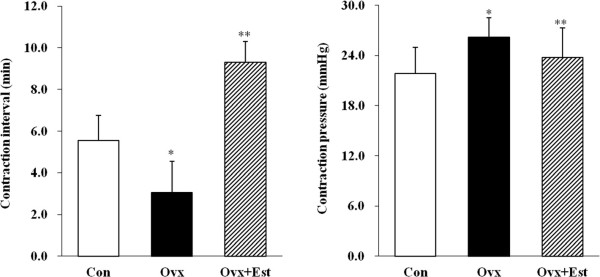
**Representative urodynamic profiles of three groups of rats: a control group (Con), a group that underwent bilateral ovariectomy (Ovx), and a group that underwent bilateral ovariectomy followed by subcutaneous injections of 17β-estradiol (Ovx?+?Est).** The contraction interval in the Ovx group was significantly shorter than that in the Con group (P?<?0.05) and was restored to the control value after estrogen treatment (P?<?0.05). Note the increased peak pressures with each voiding contraction in the Ovx group and recovery to the control value in the Ovx?+?Est group. The lower panels denote the means?±?standard errors of the mean of 5 experiments for each condition determined by cystometrogram. *P?<?0.05 vs. control, **P?<?0.05 vs. Ovx.

In cystometrograms performed 4 weeks after the operation, the contraction interval (min) was significantly lower in the Ovx group (3.1?±?1.5) than in the control group (5.6?±?1.2)(P?=?0.02), but was increased after estrogen treatment (9.3?±?1.0) (P?=?0.01). Conversely, the average contraction pressure (mmHg) was higher in the Ovx group (26.2?±?2.3) than in the control group (21.9?±?3.1)(P?=?0.03) and was decreased after estrogen treatment (23.8?±?3.5)(P?=?0.04)(Figure 1).

### Effect of estrogen on the expression of caveolin 2 and caveolin 3

**Figure 2:**
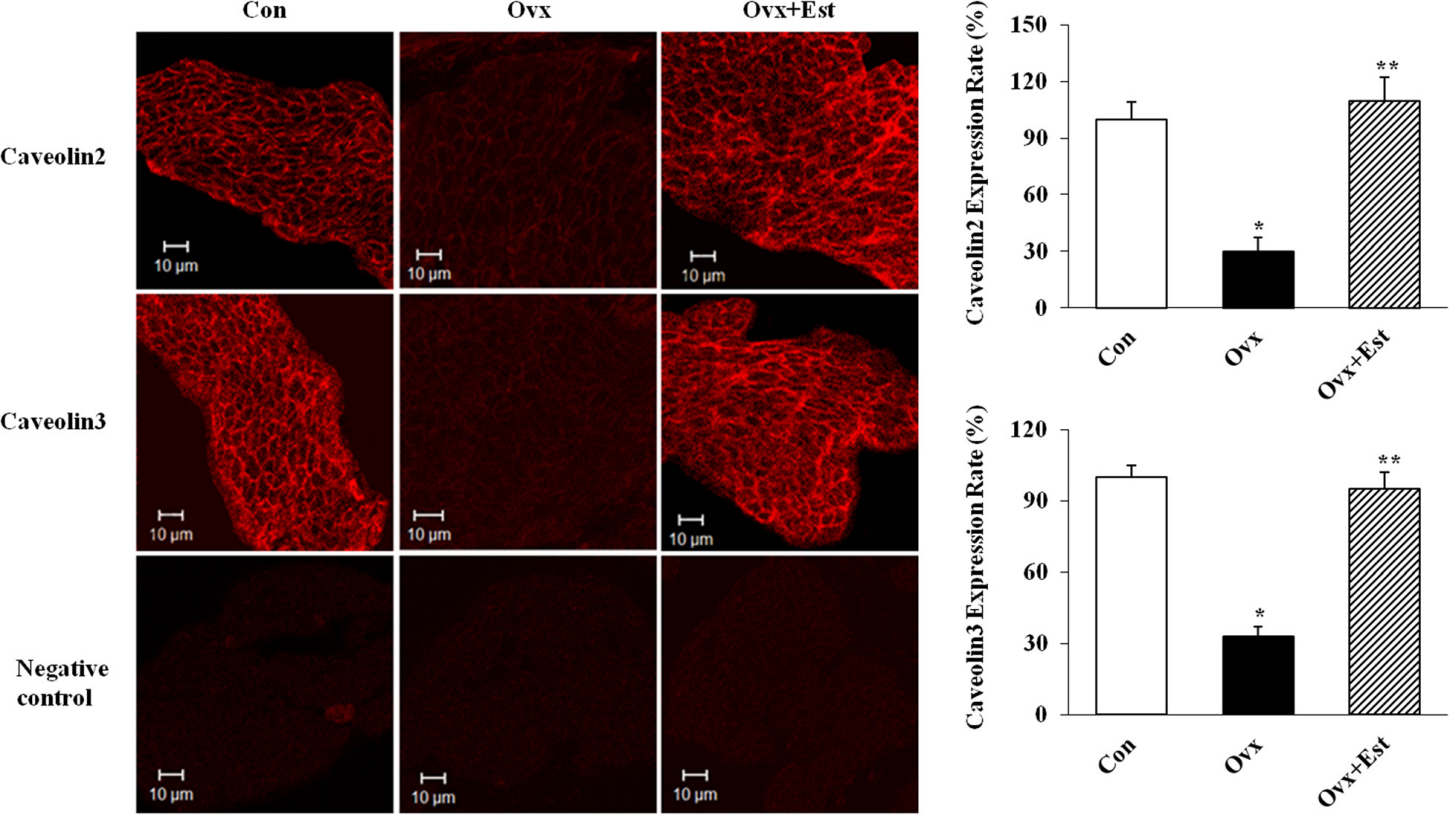
**Immunofluorescence labeling for caveolin2 and caveolin3 in rat urinary bladder.** Caveolin2 and caveolin3 expression (red) was noted throughout the cell membrane in the smooth muscle layers. Both caveolin 2 and caveolin 3 showed decreased immunoreactivity in the Ovx group but returned to the control value after estrogen treatment, as demonstrated by the Ovx?+?Est group. The horizontal scale bar at the bottom left of each figure indicates the magnification power. The right panels denote the means?±?SD of 5 experiments for each condition determined by relative densitometry. Con?=?control; Ovx?=?ovariectomy; Ovx?+?Est?=?ovariectomy plus 17β-estradiol treatment. *P?<?0.05 vs. control, **P?<?0.05 vs. Ovx.

The expression of caveolin 2 and caveolin 3 was easily detected in the smooth muscle of all groups. Caveolin 2 and caveolin 3 was mainly expressed in the cell membrane of the smooth muscle. Immunofluorescence staining showed that the Ovx group had a decreased expression of caveolin 2 (P?=?0.02) and caveolin 3 (P?=?0.01) that was restored to the control level after estrogen treatment (Ovx?+?Est group; caveolin 2, P?=?0.01; caveolin 3, P?=?0.01)(Figure 2).

**Figure 3:**
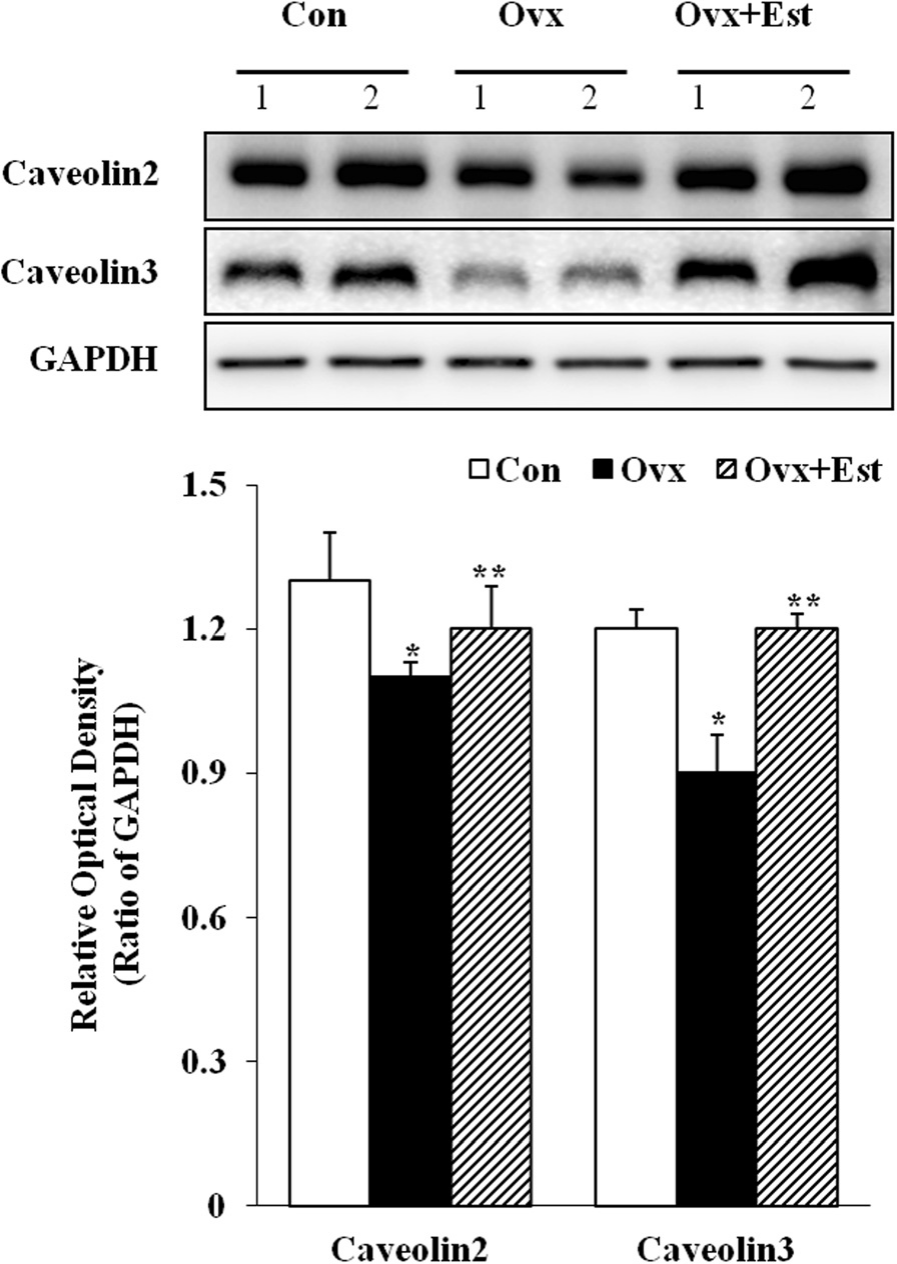
**Immunoblotting of caveolin2 and caveolin3 in the rat urinary bladder.** The anti-caveolin 2 and caveolin 3 antibodies recognized 18–20 kDa bands. Anti-GAPDH antibody recognized the 42 kDa band. Caveolin 2 and caveolin 3 protein expressions were significantly decreased in the Ovx group. However, this expression was restored to the level of the control after 17β-estradiol treatment in the Ovx?+?Est group. The lower panels denote the mean?±?standard deviation of 5 experiments for each condition determined by densitometry relative to GAPDH. Con?=?control; Ovx?=?ovariectomy; Ovx?+?Est?=?ovariectomy plus 17β-estradiol treatment; *P?<?0.05 vs. control; **P?<?0.05 vs. Ovx.

Western blot analysis revealed bands at 20 kDa corresponding to caveolin 2 and caveolin 3 proteins (Figure 3). Caveolin 2 (P?=?0.04) and caveolin 3 (P?=?0.04) protein expression was significantly lower after ovariectomy. However, the expression of these proteins was restored to the control level after 17β-estradiol treatment (caveolin 2, P?=?0.02; caveolin 3, P?=?0.01) (Figure 3).

## Discussion

In the present study, the expression of caveolin 2 and caveolin 3 was significantly decreased in the ovariectomy group but was restored to the control level after 17β-estradiol treatment. These results suggest that hormonal alteration may change the expression of caveolins in rat urinary bladder and that caveolins may play a role in the bladder dysfunction induced by ovariectomy and hormonal change.

The urinary tract is sensitive to female sex steroids and estrogen. Surgical removal of ovary and estrogen administration induced pronounced alterations in lower urinary tract structure and bladder function in an animal study [12]. Ovariectomy can induce bladder change such as decreased bladder compliance, and decreased detrusor contractility [1]. It is believed that alterations in circulating estrogen levels play a major role in mediating bladder physiology and related subjective symptoms, including urgency, frequency, and an overactive or underactive detrusor muscle. In real practice in the urologic clinic, most postmenopausal women are subject to several urologic dysfunctions, such as overactive bladder symptoms, incontinence, and urinary tract infections [13].

Caveolae has been considered to mediate several important cellular processes such as regulation of lipid transport, cholesterol homeostasis, endocytosis and transcytosis [14,15]. Recently, diverse and complex roles of caveolae have been proposed in smooth muscle cells; these roles include a site for sequestration of signaling proteins, which would facilitate, organize, and integrate responses to extracellular stimuli [5]. However, caveolins are major structural components of caveolae by bind cholesterol and promote the formation of an invaginated caveolae structure [16]. The caveolin protein family consists of three members: caveolin 1, caveolin 2, and caveolin 3. All three have been detected in smooth muscle cells.

A study conducted using caveolin 1 knockout mice provided evidence implicating caveolin in urinary bladder activity; loss of caveolin 1 impaired urinary bladder contraction and was associated with disruption of M3 muscarinic cholinergic activity in the bladder [17]. Several studies have shown that caveolae regulate bladder smooth muscle and modulate bladder contraction, and have documented pronounced alteration in caveolae under specific conditions [17-19]. Polyák et al. reported decreased numbers of caveolae in hypertrophied detrusor smooth muscle, which might contribute to alterations in signal transduction pathways that regulate the downstream effects of agonist induced bladder contraction [18]. Cristofaro et al. examined the expression of caveolin1, 2, and 3 in rat urinary bladder tissue and found reduced agonist induced bladder contraction after cholesterol depletion that was restored following cholesterol replacement [19]. The authors opined that caveolins have a central role in the regulation of receptor signaling pathways in bladder smooth muscle.

To date no study has investigated the changes in expression of caveolin 2 and 3 in bladder smooth muscle or the functional activity of these proteins in response to hormonal alteration. We already investigated the effect of hormonal alterations on the expression of caveolin 1 in the bladder smooth muscle which suggests that caveolin 1 might have a functional role in the detrusor overactivity related to hormonal alterations [6]. In the present study, the same alteration of the expression of caveolin 2 and caveolin 3 as our previous report of caveolin 1 in the smooth muscle of urinary bladder was found. It is suggested that all the three isoforms of caveolin (caveolin 1, 2, and 3) have a functional role in the overactivity of the detrusor muscle without being able to understand what is most expressed in the detrusor muscle and which may play the main functional role.

One of the possible reasons behind this influence on caveolin expression is believed to be the significance of the location of the molecules in the urinary bladder: the smooth muscle of urinary bladder is highly dependent on hormonal alterations, which induce functional change of the urinary bladder smooth muscle after estrogen deprivation.

Our results suggest that ovariectomy may lead to significant down-regulation of caveolin 2 and caveolin 3 expressions in rat urinary bladder, providing presumptive evidence that caveolins are involved in the lower urinary tract symptoms induced by hormonal alteration, probably by modification of signal transmission via caveolae in the smooth muscle. A limitation of our study is that the precise functional activity of caveolins was not fully unveiled, although we did show the clear change in expression of caveolins in the ovariectomy rat urinary bladder. Further studies are needed to investigate role of the caveolins in the underlying mechanisms of pathophysiology of bladder dysfunction after hormonal change.

## Conclusion

Hormonal alteration causes a significant change in the expression of caveolin 2 and 3 in smooth muscle of rat urinary bladder. These findings suggest that these molecules might have functional roles in the detrusor overactivity that occurs in association with hormonal alteration.

## Abbreviations

Ovx: Ovariectomy; Ovx?+?Est: Bilateral ovariectomy followed by subcutaneous injections of 17β-estradiol; GAPDH: Glyceraldehyde 3-phosphate dehydrogenase

## Competing interests

The authors declare that they have no competing interests.

## Authors’ contributions

SOK JE contributed with the conception and design of the study and drafted the manuscript, SHS collected data, evaluated the immunohistochemical stainings, performed the statistical analyses, SCL, KAC, and JSP assisted with conception and design of the study, DDK and KP conceived of the study and supervised the study and helped draft the manuscript. All authors read and approved the final manuscript.

## Pre-publication history

The pre-publication history for this paper can be accessed here:

http://www.biomedcentral.com/1471-2490/13/44/prepub
